# Medications and cognitive decline in Alzheimer's disease: Cohort cluster analysis of 15,428 patients

**DOI:** 10.1177/13872877241307870

**Published:** 2025-01-08

**Authors:** Pol Grau-Jurado, Shayan Mostafaei, Hong Xu, Minjia Mo, Bojana Petek, Irena Kalar, Luana Naia, Julianna Kele, Silvia Maioli, Joana B Pereira, Maria Eriksdotter, Saikat Chatterjee, Sara Garcia-Ptacek

**Affiliations:** 1 Division of Clinical Geriatrics, Department of Neurobiology, Care Sciences and Society (NVS), Karolinska Institutet, Stockholm, Sweden; 2 Departmenet of Medical Epidemiology and Biostatistics, Karolinska Institutet, Stockholm, Sweden; 3 Division of Neurogeriatrics, Department of Neurobiology, Care Sciences and Society (NVS), Karolinska Institutet, Stockholm, Sweden; 4 Faculty of Medicine, University of Ljubljana, Ljubljana, Slovenia; 5 Clinical Institute of Genomic Medicine, University Medical Centre Ljubljana, Ljubljana, Slovenia; 6 Department of Neurology, University Medical Centre Ljubljana, Ljubljana, Slovenia; 7 Team Neurovascular Biology and Health, Clinical Immunology, Department of Laboratory Medicine, Karolinska Institutet, Stockholm, Sweden; 8 Neuro Division, Department of Clinical Neurosciences, Karolinska Institutet, Stockholm, Sweden; 9 Aging and Inflammation Theme, Karolinska University Hospital, Stockholm, Sweden; 10 School of Electrical Engineering and Computer Science, KTH Royal Institute of Technology, Stockholm, Sweden

**Keywords:** Alzheimer's disease, cohort study, comorbidity, metformin, Mini-Mental State Examination, oxazepam, pharmacological treatments, statins, warfarin, zopiclone

## Abstract

**Background:**

Medications for comorbid conditions may affect cognition in Alzheimer's disease (AD).

**Objective:**

To explore the association between common medications and cognition, measured with the Mini-Mental State Examination.

**Methods:**

Cohort study including persons with AD from the Swedish Registry for Cognitive/Dementia Disorders (SveDem). Medications were included if they were used by ≥5% of patients (26 individual drugs). Each follow-up was analyzed independently by performing 100 Monte-Carlo simulations of two steps each 1) k-means clustering of patients according to Mini-Mental State Examination at follow-up and its decline since previous measure, and 2) Identification of medications presenting statistically significant differences in the proportion of users in the different clusters.

**Results:**

15,428 patients (60.38% women) were studied. Four clusters were identified. Medications associated with the best cognition cluster (relative to the worse) were atorvastatin (point estimate 1.44 95% confidence interval [1.15–1.83] at first follow-up, simvastatin (1.41 [1.11–1.78] at second follow-up), warfarin (1.56 [1.22–2.01] first follow-up), zopiclone (1.35 [1.15–1.58], and metformin (2.08 [1.35–3.33] second follow-up. Oxazepam (0.60 [0.50–0.73] first follow-up), paracetamol (0.83 [0.73–0.95] first follow-up), cyanocobalamin, felodipine and furosemide were associated with the worst cluster. Cholinesterase inhibitors were associated with the best cognition clusters, whereas memantine appeared in the worse cognition clusters, consistent with its indication in moderate to severe dementia.

**Conclusions:**

We performed unsupervised clustering to classify patients based on their current cognition and cognitive decline from previous testing. Atorvastatin, simvastatin, warfarin, metformin, and zopiclone presented a positive and statistically significant associations with cognition, while oxazepam, cyanocobalamin, felodipine, furosemide and paracetamol, were associated with the worst cluster.

## Introduction

Persons with Alzheimer's disease (AD) often have additional comorbidities.^1^ Medications such as metformin and thiazolidinediones, for type 2 diabetes mellitus, statins for hypercholesterolemia or antihypertensives, have shown beneficial effects on dementia risk reduction, although their cognitive effects in persons with established dementia is unclear.^2–6^ There is a consensus that antipsychotics have negative effects on cognition and survival in patients with dementia and Swedish guidelines state that they should be used to treat psychotic symptoms only when other interventions have failed.^7–9^ Treatment for comorbidities can improve the trajectory of dementia patients, although it is unclear whether these cognitive improvements are solely due to indirect factors, such as overall better health, or direct cognitive effects from these medications prescribed for other conditions.^10–13^

In healthcare, vast amounts of data are collected, holding hidden patterns and relationships essential for addressing challenges in the field. Unsupervised clustering techniques serve as a powerful method to reveal these hidden relationships and patterns without requiring predefined labels.^14–16^ This approach not only identifies natural groupings among selected variables but also allows for exploring connections between these clusters and other variables. The combination of clustering with statistical tests provides robust tools for scrutinizing relationships within the identified clusters.^17–19^ This holistic approach enhances our understanding of latent dynamics in healthcare datasets, offering valuable insights for navigating healthcare challenges.^20–22^

The objective of this study was to investigate the patterns of the relationship between medications and cognitive decline in patients with AD, measured as decline in Mini-Mental State Examination (MMSE) over time. For this study, we included patients from the Swedish Register of Cognitive Diseases and Dementia—SveDem—who had been diagnosed with either AD or mixed dementia (MD). The definition of MD encompassed persons who suffer from AD along with any other type of dementia, typically vascular dementia: the combination of AD and MD in Sweden probably best represents what is epidemiologically considered AD dementia in international cohorts, so we combined these two groups for this study.^23^ Our study aimed to screen a wide range of frequently used medications, irrespective of indication. We conducted a cohort study using SveDem to 1) implement an unsupervised clustering to classify patients into distinct groups based on their cognitive progression across follow-ups, 2) analyze the variations of medication distribution between clusters with the aim to detect medications that may positively or negatively affect cognition.

## Methods

### Data sources

This study integrated data from two different Swedish registers. The main register used in the study was SveDem, a national quality register established on May 1, 2007, with the objective of monitoring and following up all dementia patients in Sweden.^24^ Individuals are registered in SveDem at the time of diagnosis and then followed-up every 9 to 15 months.^25^ SveDem includes patient demographics, type of dementia disorder, MMSE, among other variables. Drug usage information was obtained from the Swedish Prescribed Drug Registry (PDR), which contains records of all prescribed drugs dispensed in Sweden since 2005 and has almost 100% coverage.^26^ The data of the two was linked through the unique personal identity numbers and then pseudonymized.

### Study population

**Figure 1.**
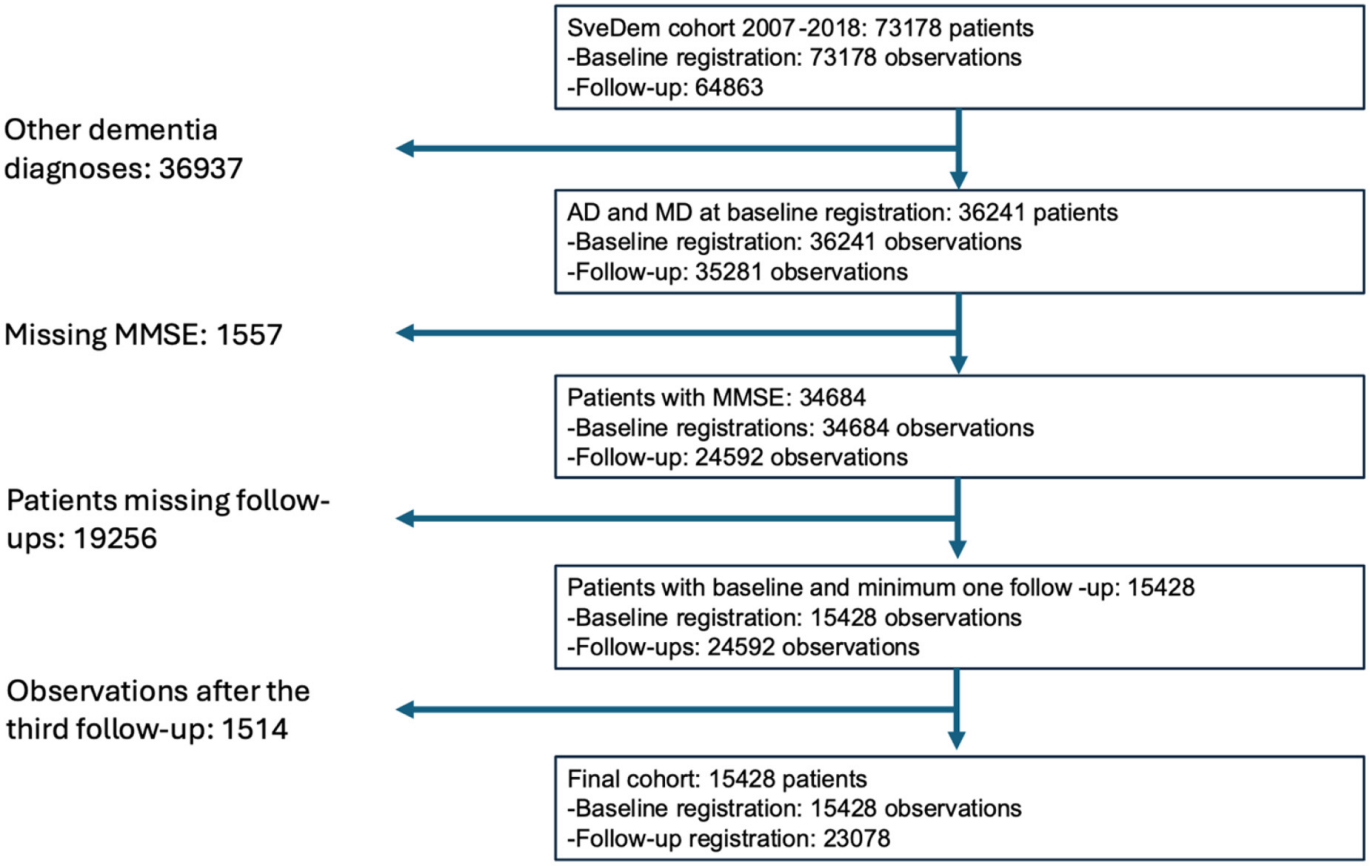
**Selection flowchart of SveDem patients included in the study, from registrations between May 2007 and December 2018.** The final number of included patients is 15,428, with 15,428 baseline and 23,078 follow-up registrations. AD: Alzheimer's disease; MD: mixed dementia; MMSE: Mini-Mental State Examination.

This patient cohort included AD and MD patients whose data was recorded between May 1, 2007, and October 16, 2018. Patients were included if they had a minimum of two entries in SveDem, including baseline and at least one follow-up, with MMSE to calculate cognitive decline trajectories. Figure 1 shows the patient selection flowchart.

### Exposures and outcome

Medications were extracted from the PDR with their corresponding Anatomical Therapeutic Chemical (ATC) codes and recorded as binary variables. The selection was based on pharmacy dispensation records within the previous 100 days preceding a baseline or follow-up registration. We limited the inclusion to medications that appeared in more than 5% of all observations in our cohort. Twenty-six medications met this inclusion criteria and are summarized in Supplemental Table 1. MMSE scores and MMSE decline with respect to the previous visit, were the two variables used as outcomes for patient clustering.

### Clustering approach

**Figure 2.**
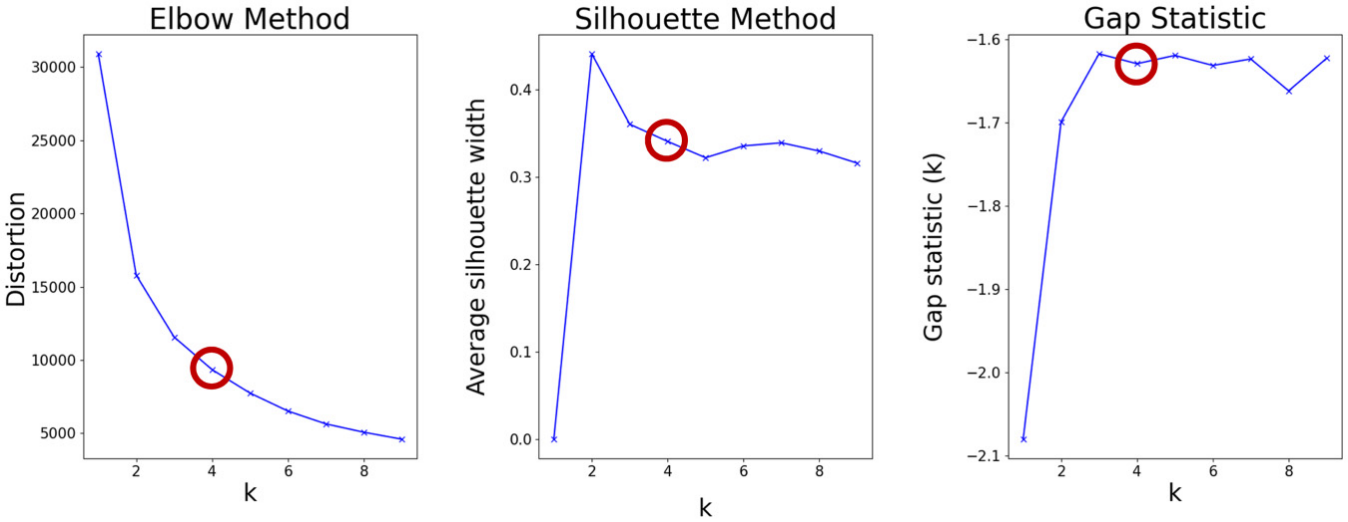
**Plot of methods to determine optimal number of clusters.** Plot of Elbow method (on the left), Silhouette score method (on the middle) direct methods, and gap statistic method (on the right) to determine the optimal number of clusters in K-means algorithm, for a single simulation on the first follow-up data. Silhouette and gap statistics methods could lead to a selection of 2 and 3 clusters respectively, but Elbow method leads towards a higher cluster number. The selection of 4 clusters is compatible with the three methods implemented.

Cluster analysis was conducted on each follow-up observation independently of other time-points. The objective was to cluster patients into separate groups based on their cognitive differences at each time point. The clustering variables were the patient's current cognition, as measured by MMSE score, and MMSE score decline from the prior measure. The K-Means clustering algorithm was chosen due to the simplicity of the two-dimensional space created by the two variables and the need for visual interpretability of the clusters. The optimal number of clusters was determined by Elbow, Silhouette statistic and gap statistic methods^27^ (Figure 2). Supplemental Figure 1 illustrates the distribution density of patients in the SveDem registry on the delimited 2D map. To account for stability, consistency, and further generalization, we implemented Monte-Carlo simulations and averaged the results. The code to carry out the analysis was implemented in Python, using libraries such as *pandas, sklearn, scipy,* and *statsmodels*, and it is available on github.com (https://github.com/Polgrauj/KI-Clustering-Drug-Project).

### Statistical analysis

To compare medication distribution between clusters, Z-test of proportions was employed, using a cluster-wise approach. For each pair-wise cluster analysis, Benjamini-Hochberg procedure was used to control the false discovery rate (FDR) (q < 0.05). The confidence intervals for the proportions in the clusters were computed by implementing Fisher's exact test using *scipy* library.

The experiment was run through multiple simulations to ensure convergence of the variance of each medication's Z-scores close to the true value. The optimal number of simulations was calculated according to

$$N={\left(\frac{CL\cdot \sigma }{\emptyset }\right)}^{2},$$

where σ is the sample standard deviation, CL is the confidence level set to 99% ( CL=2.576 ), and ∅ corresponds to the level of precision, which is the difference between the sample mean ( $\bar{x}$ ) and the true population mean ( $\mu_{x}$ ),^28^ set to ∅=0.1 .

After running all simulations, we considered statistically significant drugs those exhibiting a p_value<0.05 ( $|Z_{score}|\geq 1.96$ ) after FDR correction in at least 80% of Monte-Carlo simulations.

### Ethical considerations

This study was approved by the Swedish Ethical Review Authority with the reference number (#2021-0043) and was performed following the guidelines in the Declaration of Helsinki.

## Results

### Cohort characteristics

SveDem comprised a total of 138,041 observations from 73,178 patients between May 1, 2007, and December 31, 2018. After filtering for AD patients with baseline MMSE and one or more follow-ups with MMSE, we included a final cohort of 15,428 patients (60.38% women; mean [SD] age at diagnosis, 77.29 [7.59] years) with a total of 38,506 registrations. The selection process flowchart is illustrated in Figure 1 and patient characteristics in Table 1. Among the included patients, 9980 (64.68%) had only one follow-up registered, 3246 (21.03%) had 2 follow-ups, and 2202 (14.29%) had 3 or more follow-ups. The mean [standard deviation-SD] time elapsed between the baseline registration and each follow-up was 379.55 [273.59] days for the first, 803.25 [360.77] for the second, and 1208.60 [392.57] for the third follow-ups.

**Table 1.** Characterization at baseline and three first follow-ups of included patients.

SveDem	Baseline	Follow-up 1	Follow-up 2	Follow-up 3
Patients	15428	15428	5448	2202
Total follow-ups:				
- 1	9980			
- 2	3246	—-	—-	—-
- 3	1296	—-	—-	—-
- > 3	906			
Gender (Females)	9316 (60.38%)	9316 (60.38%)	3155 (57.91%)	1256 (57.04%)
Age at diagnosis (mean [SD])	77.30 [7.60]	77.30 [7.60]	75.60 [7.85]	74.62 [7.85]
Age (mean [SD])	77.30 [7.60]	78.33 [7.59]	77.80 [7.87]	77.94 [7.87]
Diagnosis:				
- AD	10474 (67.89%)	10306 (66.79%)	3963 (72.74%)	1664 (75.56%)
- Mixed Dementia	4954 (32.11%)	4949 (32.08%)	1369 (25.13%)	477 (21.66%)
- Other (Change on follow-up)	—-	173 (1.13%)	116 (2.13%)	61 (2.78%)
Residency:				
- Ordinary	15114 (97.96%)	14415 (93.43%)	5082 (93.28%)	2030 (92.19%)
- Special, temporary	98 (0.64%)	167 (1.08%)	67 (1.23%)	29 (1.32%)
- Special, permanent	158 (1.02%)	500 (3.24%)	146 (2.68%)	67 (3.04%)
- Special adapted for dementia, permanent	39 (0.25%)	319 (2.07%)	144 (2.64%)	72 (3.27%)
- Do not know	19 (0.13%)	27 (0.18%)	9 (0.17%)	4 (0.18%)
Days elapsed from the diagnosis (mean [SD])	—-	379.55 [273.59]	803.25 [360.77]	1208.60 [392.57]
Days elapsed from the previous follow-up (mean [std])	—-	379.55 [273.59]	432.88 [237.41]	423.69 [201.39]
MMSE Score (mean [SD])	22.29 [4.27]	20.79 [5.04]	19.83 [5.37]	19.34 [5.60]
MMSE Decline (mean [SD])	—-	−1.50 [3.93]	−2.22 [3.71]	−2.18 [3.43]
Global estimate of cognition				
- Improved		1884 (12.21%)	290 (5.32%)	84 (3,81%)
- Unchanged		6706 (43.47%)	2091 (38.38%)	848 (38.51%)
- Deteriorated		6351 (41.17%)	2870 (52.68%)	1193 (54.18%)
- Do not know	—-	411 (2.66%)	158 (2.90%)	56 (2.54%)
- Missing		76 (0.49%)	39 (0.72%)	21 (0.96%)

SD: standard deviation; AD: Alzheimer's disease; MMSE, Mini-Mental State Examination

Cognition was measured with MMSE scores and MMSE decline at each follow-up. The mean MMSE score at baseline was 22.29 (standard deviation-SD 4.27). For the follow-ups, the MMSE score and decline were: 20.79 (5.05) MMSE score and −1.50 (3.93) decline at the first follow-up, 19.83 (5.37) and −2.22 (3.71) at the second, and 19.34 [5.60] and −2.18 [3.43] at the third follow-up, considering remaining patients.

### Characterization of the patient clusters

**Figure 3.**
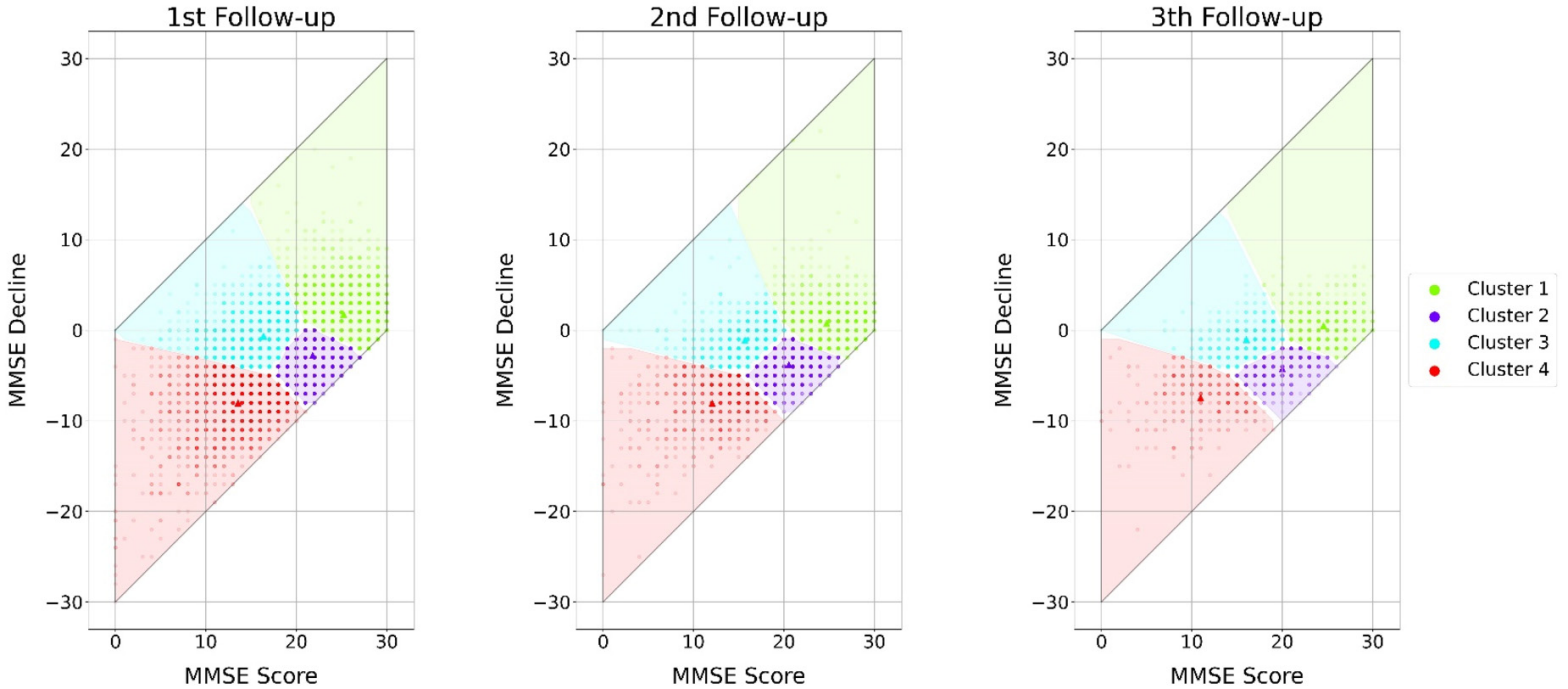
**Single Monte-Carlo simulation clustering results for the three follow-ups analyzed.** MMSE score at the time of the follow-up on the X-axis, and MMSE decline as the MMSE difference experienced with respect the previous visit, on the Y-axis. Scattered points represent patients, with darker points representing the largest number of patients. Cluster centroids are represented by a dark triangle, and clusters’ covering area delimited by the plotted surface. Clusters are labeled as: (1 – green) high MMSE score with improvement or no decline, (2 – purple) high MMSE with cognitive decline, (3 – blue) low MMSE with variable decline, and (4 – red) high decliners. MMSE: Mini-Mental State Examination.

The optimal number of clusters was four. The elbow, silhouette score, and gap statistic methods supported this conclusion, as shown in Figure 2. The four identified clusters characterized the patients based on their cognition status, determined by their MMSE scores and decline, depicted in Figure 3. The clusters are divided into: high MMSE-score without cognitive decline (1), high MMSE-score with cognitive decline (2), low MMSE-score without cognitive decline (3), and low MMSE-score with cognitive decline (4). Supplemental Figure 2 and Supplemental Table 2 characterize the clusters on each follow-up by the clustering variables (MMSE score and decline) and the days from dementia diagnosis. Patients in the worst cluster in terms of cognitive performance (4) underwent follow-up assessments later, on average than those in the other clusters (mean [SD], 512.65 [404.30] days on compared to 340.01 [214.72], 392.67 [289.99], and 354.50 [217.36] on the first, second and third clusters, respectively), and this difference is preserved for the upcoming follow-ups. Additionally, this cluster had a smaller number of patients than the other three, particularly in the first follow-up (1915 compared to 6031, 4465, and 3017 for the first, second and third clusters respectively).

### Identification of medications statistically significantly associated with the clusters

**Figure 4.**
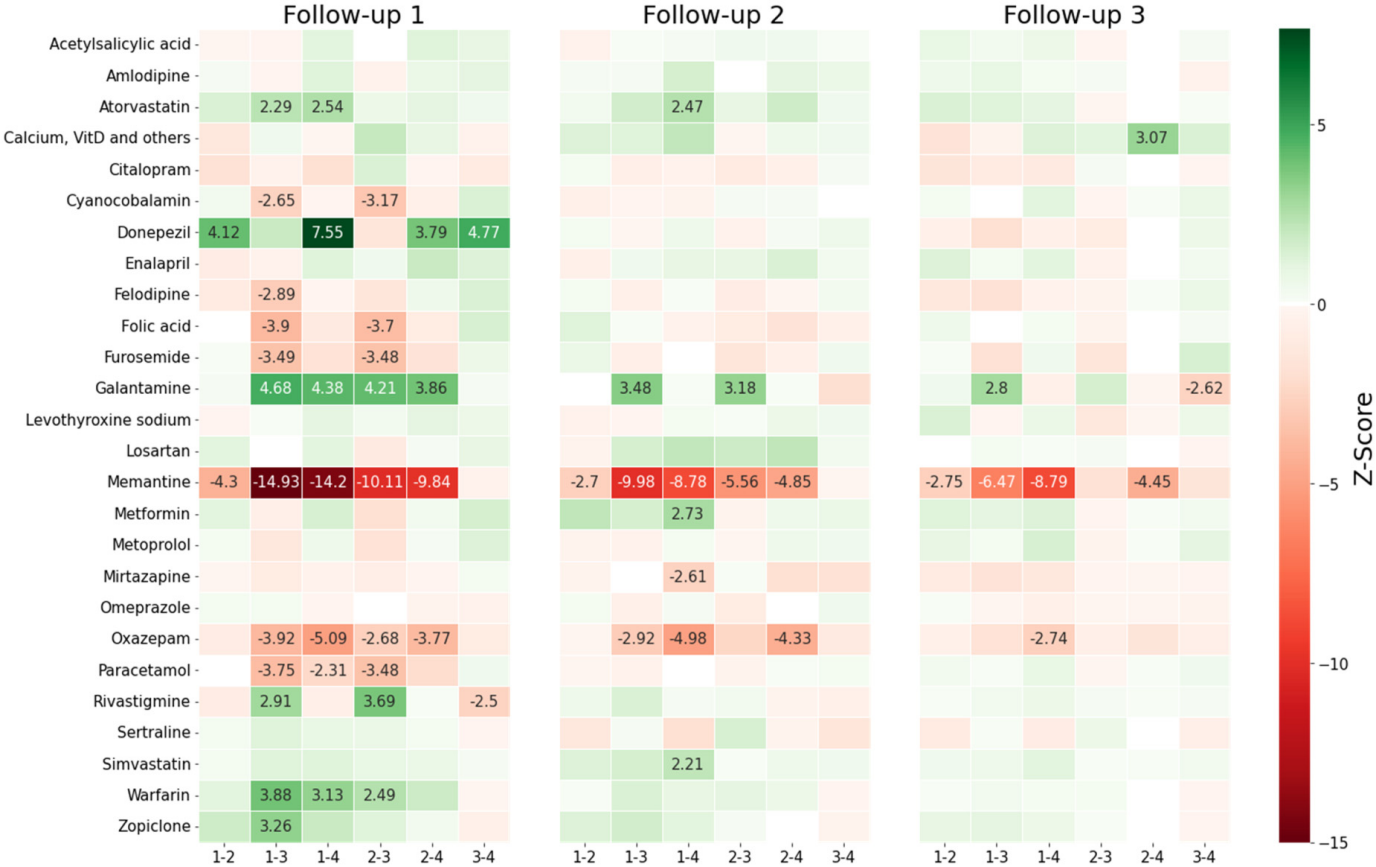
Heatmap of the averaged Z-scores obtained from implementing the proportions tests to each cluster combination for the 3 first follow-ups. The test is simulated 100 times and Z-scores are averaged. A positive averaged Z-score (green) corresponds to a greater proportion of medication users on the cluster indicated on the left, and a negative Z-score (red) corresponds to a greater proportion on the cluster on the right. Z-scores means are only annotated for those drugs that appeared relevant (p-value < 0.05 in minimum 80% of the simulations after accounting for FDC). A Z-score >|±1.95| corresponds to a p-value < 0.05. Extreme values (darker colors) correspond to a smaller p-value and therefore higher statistical confidence.

The statistical analysis revealed significant differences in medication usage between clusters at all follow-up time points, but particularly in the first two. We determined that 100 Monte-Carlo simulations were sufficiently representative based on the previously described methodology. Figure 4 presents the cluster-wise results of this analysis as a heatmap. Supplemental Table 3 presents the same results numerically, showing p-values and odds ratio point estimates together with 95% confidence intervals on each follow-up for the statistically significant drugs. Odds ratio of point estimates (PE) with their confidence intervals (CIs) represent the probabilities of belonging to one cluster compared to another regarding dispensation of the specified drug.

Figure 4 shows the comparison of the cluster with high MMSE score without cognitive decline (1) in relation to the three other clusters. The four dementia medications were those which presented the strongest statistical significance (indicated by Z-scores) across all clusters. The three cholinesterase inhibitors (ChEIs) were associated with clusters with better cognition. Memantine was associated with less frequent prescription in the best cluster, for the first follow-up comparisons of clusters 1 versus 3: showed a point estimate (PE) of 0.45; 95% confidence interval (95% CI) 0.40–0.50.

Other medications not prescribed for dementia also presented associations with cognitive clustering. Warfarin (clusters 1 versus 3: PE 1.58 95% CI [1.28–1.97]), atorvastatin (clusters 1–3: 1.30 [1.08–1.58]) and zopiclone (clusters 1–3: 1.35 [1.15, 1.58]) were associated with the high cognitive performance cluster at first follow-up. Again atorvastatin (cluster 1 versus 4: 1.76 [1.20–2.64]), together with simvastatin (cluster 1 versus 4: 1.41 [1.11–1.78]), and metformin (1–4: 2.08 [1.35–3.33]) were more frequent in the high cognitive performance cluster in the second follow-up. Medications which were more frequent in low cognitive performance clusters in the first follow-up were cyanocobalamin (cluster 1 versus 3: 0.86 [0.78–0.95]), felodipine (1–3: 0.79 [0.66–0.95]), folic acid (1–3: 0.74 [0.65–0.84]), furosemide (1–3: 0.76 [0.66–0.89]), and paracetamol (1–3: 0.79 [0.70–0.88]). Oxazepam was negatively associated with cognition in all three follow-ups, while mirtazapine was negatively associated with cognition in the second follow-up (clusters 1 versus 4: 0.66 [0.51–0.87]).

An examination of drug-response effects exceeds the objectives of this study. For illustrative purposes and to aid interpretation, we present descriptive statistics for the Defined Daily Doses (DDDs) for patients treated with specific drugs at each MMSE follow-up (Supplemental Table 4). As an example, for patients taking galantamine the median DDD was 1 (IQR 0.42) where 1 DDD represents 16 mg, while patients taking rivastigmine took a median of 0.9 DDD (0.54) with 1 DDD equals 9 mg of per oral or 9.5 mg transdermal rivastigmine. The median dose of oxazepam (0.1 DDD; IQR 0.15) corresponds to 5 mg of oxazepam since 1 DDD equals 50 mg.

## Discussion

In this cohort study from SveDem, we conducted a clustering analysis of patients with AD or MD according to MMSE score and MMSE decline from prior registration. We performed over 100 Monte-Carlo simulations, providing statistical stability. Then we identified the medications that exhibited significant differences in usage between the cluster of patients characterized by high cognition and no cognitive decline compared to the other clusters. Atorvastatin, simvastatin, warfarin, metformin, cholinesterase inhibitors, and zopiclone were associated with good cognition clusters, while oxazepam, paracetamol, cyanocobalamin, felodipine, memantine, and furosemide were associated with poor cognition clusters.

Naturally, the medications prescribed for dementia presented the strongest associations with the clusters, as indicated by Z-scores (Figure 4). The three ChEIs were associated with clusters with better cognition, in concordance with their results from clinical trials and prior research in SveDem.^29^ The official indication of ChEIs is mild to moderate dementia. Swedish guidelines recommend cholinesterase inhibitors in mild to moderate AD or mixed dementia with high priority and recommend adding memantine to ChEIs in severe dementia.^9,29–32^ Memantine was associated with less frequent prescription in the best cluster, which is reasonable, since the prescription indication is for moderate to severe AD.^33,34^ This study also detected other medications not prescribed for dementia, which were associated with cognitive clustering: warfarin, atorvastatin, and zopiclone were associated with the high cognitive performance cluster at first follow-up, while again atorvastatin together with simvastatin, and metformin were more frequent in the high cognitive performance cluster in the second follow-up.^9^ Medications which were more frequent in low cognitive performance clusters were oxazepam (in all three follow-ups), cyanocobalamin, felodipine, folic acid, furosemide, and paracetamol (in the first follow-up). Mirtazapine was associated with worse cognition clusters in the second follow-up.

Previous studies^35–37^ have associated the use of statins with a decreased risk of dementia. Various meta-analyses and cohort studies have reported the safety of statins in relation to cognitive decline,^37–40^ and statins have been proposed as treatments for AD, although clinical trials failed.^2,4–6,41^ A recent study from our group found an association between statins and better cognitive outcomes in AD.^2^ Our findings may suggest a positive effect of statins in cognition, but further studies are necessary to replicate this finding. Felodipine was more frequent in low cognitive clusters, although studies focusing on the effects of felodipine treatment in the dementia population are rare and have reported conflicting results.^10,42^ In our previous work in AD patients in SveDem, felodipine was associated with higher mortality risk.^10^

The anticoagulant medication warfarin was more used in the first cluster. It has been suggested that warfarin is associated with reduced risk of dementia for patients with atrial fibrillation,^43^ although non-vitamin K oral anticoagulants have shown even lower risk.^44,45^ A previous study by our group also supports the use of warfarin in appropriate cases in patients with atrial fibrillation and dementia.^46^ Metformin, a first-line treatment for diabetes mellitus type 2, presented double the frequency of use in the cluster with the best cognition compared to the worst. Long-term use of metformin has been associated with possible neuroprotective effects in previous studies.^47–49^ The clinical trial MET-FINGER is currently investigating metformin as a therapeutic agent to prevent dementia in at-risk subjects.^50^

The use of insomnia medications such as zopiclone and oxazepam for elderly patients with dementia is a topic of ongoing discussion in terms of weighing their risks and benefits, and only short-term use is recommended.^51–54^ In one cohort study, lower doses of zopiclone somewhat mitigated the risks.^51^ In contrast, oxazepam was more frequent in patients with low MMSE scores and significant cognitive decline in all follow-ups, with high differences in proportions by doubling the frequency on the worst cognition cluster. These results are in accordance with the main dementia guidelines and different studies discouraging its use in the older population and dementia patients.^55–59^

Our results shed insights into the association between the most prescribed drugs in Sweden and cognitive decline in persons with AD and MD. While medications prescribed specifically for dementia treatment aligned with our initial expectations, we also observed surprising differences in the association between cognition and the consumption of medications for other comorbidities. These findings highlight potential candidates for medication repurposing for AD, including statins, warfarin, zopiclone and metformin, which should be examined in future studies. Conversely, oxazepam is strongly associated with cognitive deterioration, consistent with its sedative effect, and our findings support restricting its use in AD as already stated in treatment guidelines.

### Strengths and limitations

To the best of our knowledge, this study is the first study to investigate the relationships between a wide range of commonly prescribed medications and the cognition of patients with AD and MD using a real-world clinical quality registry. Our analysis was conducted using SveDem, the largest quality dementia registry of its kind in the world. The use of this dataset allowed us to examine the drug consumption patterns on a substantial portion of Swedish dementia population under real-life conditions. The use of this dataset strengthens the generalizability of our findings.

Another strength of this study is the rigorous criterion we employed to determine the statistical significance of the drugs. In addition to controlling for the chance of false positives using false discovery rate corrections, we performed 100 Monte-Carlo simulations and considered drugs to be statistically significant only if they met the significance threshold in at least 80% of the simulations. This conservative criterion enhances confidence in our findings; however, it is important to note that it may lead to the underestimation of the importance of certain medications.

Despite these strengths, our study has some limitations. Firstly, it is not designed to establish causality or directly attribute the observed statistical differences to the effect of medication on cognition. Hence, it must be interpreted as a screening exercise to identify medications for future research. Secondly, our cohort experiences an exponential decay of patients across follow-ups, due to mortality, drop-out, moving to special accommodation, and other reasons,^24^ limiting our ability to obtain results in later follow-ups. Because of the large number of drugs examined, we did not evaluate drug dosages, which is an important aspect to consider in future research. As is shown in the summary statistics of medication doses presented in Supplemental Table 4, dispensed doses may have contributed to some of our results, e.g., simvastatin (median 0.7 mg; IQR 0.67) where 1 DDD equals 30 mg appears to be dispensed at lower doses than the more potent atorvastatin (median 1.8 mg; IQR 1; 1 DDD = 20 mg).

### Future work

This study focused on analyzing each follow-up separately, i.e., capturing snapshots at specific timepoints. However, to gain a comprehensive understanding of the overall cognition trajectory related to medications, future research should include a longitudinal study design.

Furthermore, the clustering approach employed in this study utilized a hard clustering method, where each patient is assigned to only one cluster at a time. To further explore the relationships between medication use and cognitive profiles, future investigations should consider implementing a soft clustering approach with drug weighting across clusters. This would enable a more nuanced understanding of the drug proportions within each cluster, facilitating a more comprehensive analysis of the associations.

Lastly, our findings suggest that certain drugs, such as statins, metformin, zopiclone or warfarin, may have positive effects on cognition. Thus, it would be valuable to conduct further research specifically targeting the direct effects of these drugs on the cognition of patients with AD.

### Conclusions

In this unsupervised clustering study, we clustered 15,428 AD patients into four different groups and identified the drugs presenting significant between-group differences. Atorvastatin, warfarin, zopiclone, simvastatin, and metformin were significantly more frequent among the best cognition cluster of patients. Oxazepam was found to be more common in patients in the low MMSE clusters for all follow-ups, while cyanocobalamin, felodipine, folic acid, furosemide, paracetamol, and mirtazapine were overrepresented in the low cognition clusters in a single follow-up. Future research should address whether these medications are detrimental to patients with AD.

## Supplemental Material

sj-docx-1-alz-10.1177_13872877241307870 - Supplemental material for Medications and cognitive decline in Alzheimer's disease: Cohort cluster analysis of 15,428 patients Supplemental material, sj-docx-1-alz-10.1177_13872877241307870 for Medications and cognitive decline in Alzheimer's disease: Cohort cluster analysis of 15,428 patients by Pol Grau-Jurado, Shayan Mostafaei, Hong Xu, Minjia Mo, Bojana Petek, Irena Kalar, Luana Naia, Julianna Kele, Silvia Maioli, Joana B Pereira, Maria Eriksdotter, Saikat Chatterjee and Sara Garcia-Ptacek in Journal of Alzheimer's Disease
