# Supplementary material for: Medications and cognitive decline in Alzheimer's disease: Cohort cluster analysis of 15,428 patients
Source: J Alzheimers Dis. 2025 Jan 8;103(3):931–40. doi: 10.1177/13872877241307870 (PMC13066463; doi:10.1177/13872877241307870)
Supplement: sj-docx-1-alz-10.1177_13872877241307870 - Supplemental material for Medications and cognitive decline in Alzheimer's disease: Cohort cluster analysis of 15,428 patients [file sj-docx-1-alz-10.1177_13872877241307870.docx]

**Supplemental Material**

**Medications and cognitive decline in Alzheimer’s disease: Cohort cluster analysis of 15,428 patients**

**Supplemental Table 1**. Drugs included in the study with their corresponding ATC code and percentage of users.

| **Drug name** | **ATC codes** | **Cohort percentage** | **Indication** |
| --- | --- | --- | --- |
| Donepezil | N06DA02 | 30,96 | Dementia (ChEIs) |
| Acetylsalicylic acid | B01AC06 | 28,88 | Analgesic, antipyretic, anti-inflammatory |
| Cyanocobalamin | B03BA01 | 25,44 | B12 vitamin |
| Simvastatin | C10AA01 | 19,64 | Cholesterol lowering |
| Paracetamol | N02BE01 | 17,75 | Analgesic and antipyretic |
| Memantine | N06DX01 | 17,31 | Dementia (NMDA receptor antagonist) |
| Galantamine | N06DA04 | 16,32 | Dementia (ChEIs) |
| Metoprolol | C07AB02 | 15,53 | Cardiac diseases (beta blocker) |
| Folic acid | B03BB01 | 13,44 | B9 vitamin |
| Citalopram | N06AB04 | 12,81 | Antidepressant |
| Enalapril | C09AA02 | 12,58 | Hypertension (ACE inhibitor) |
| Rivastigmine | N06DA03 | 11,08 | Dementia (ChEIs) |
| Levothyroxine sodium | H03AA01 | 10,55 | Hyperthyroidism |
| Omeprazole | A02BC01 | 10,18 | Stomach acidity |
| Calcium, combinations with vitamin D and/or other drugs | A12AX | 10,15 | Vitamins |
| Amlodipine | C08CA01 | 9,87 | Hypertension (CCB) |
| Zopiclone | N05CF01 | 9,64 | Insomnia |
| Furosemide | C03CA01 | 8,57 | Diuretic / Hypertension |
| Mirtazapine | N06AX11 | 8,23 | Antidepressant |
| Oxazepam | N05BA04 | 6,67 | Anxiolytics |
| Felodipine | C08CA02 | 6,48 | Hypertension (CCB) |
| Atorvastatin | C10AA05 | 6,22 | LDL cholesterol |
| Metformin | A10BA02 | 5,53 | Antidiabetic |
| Warfarin | B01AA03 | 5,46 | Anticoagulant |
| Losartan | C09CA01 | 5,35 | Hypertension (ARB) |
| Sertraline | N06AB06 | 5,23 | Antidepressant |

We screened the 100 most prescribed drugs in Sweden and only included those that were present in at least 5% of patients in the study cohort. ATC: Anatomical Therapeutical Chemical code; ChEIs: cholinesterase inhibitors; NMDA: N-Methyl-D-aspartate; ACE: angiotensin-converting enzyme; CCB: calcium channel blocker; LDL: low-density lipoprotein; ARB: angiotensin receptor blockers

**Supplemental Figure 1.** Mini-Mental State Examination (MMSE) distribution map of the SveDem Alzheimer’s disease and mixed dementia patients included in the study for the first three follow-ups.


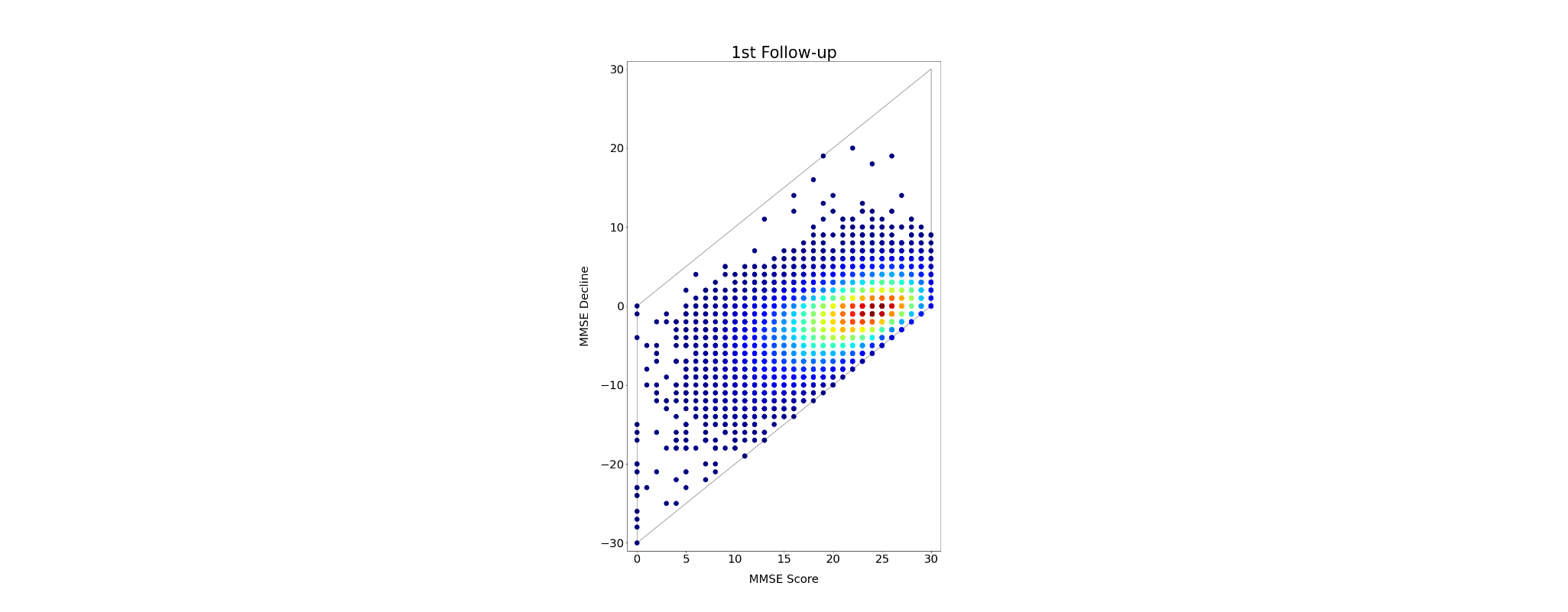

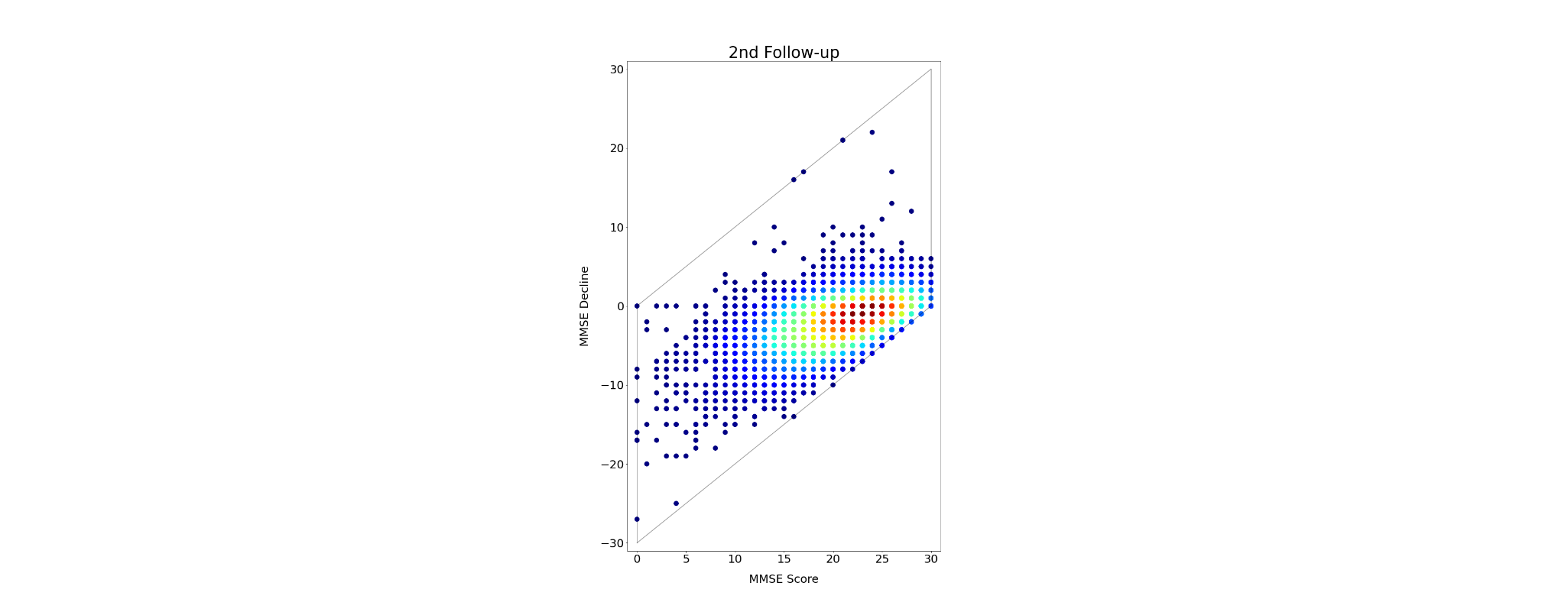

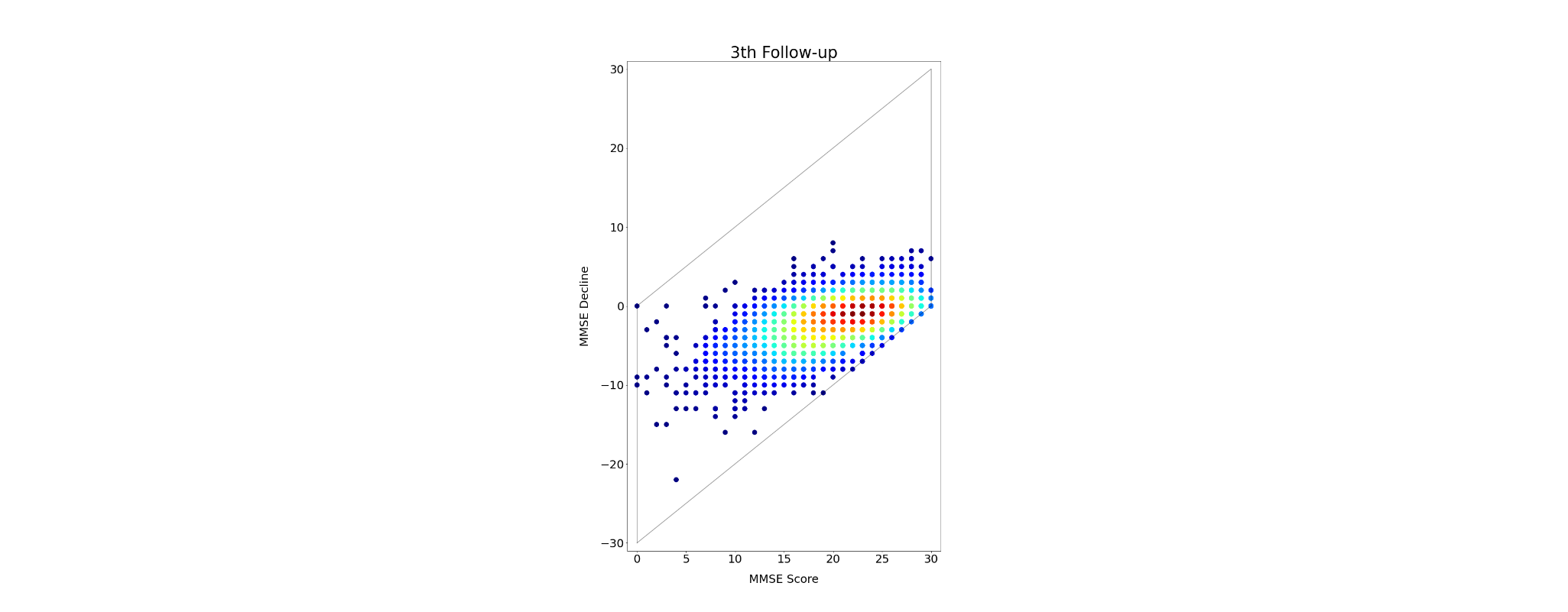


Cognition is represented by the measured MMSE score at the time of the follow-up on the X-axis, and MMSE decline as the MMSE difference experienced with respect the previous visit, on the Y-axis. The trapezoid drawn in black represents the area of compatible values.


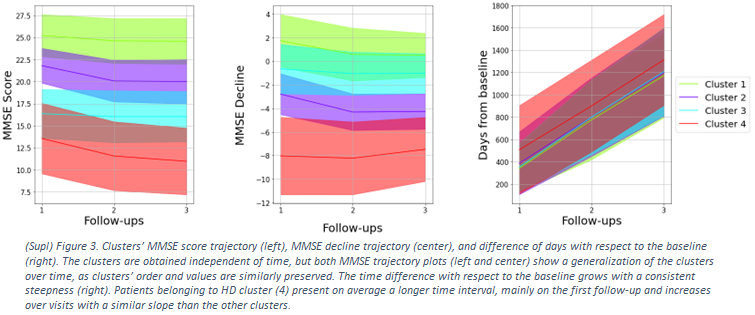
**Supplemental Figure 2.** Mini-Mental State Examination (MMSE) score and decline trajectories for the different clusters

MMSE score trajectory (left), MMSE decline trajectory (center), and difference of days with respect to the baseline (right) for the different clusters. The clusters are obtained independent of time, but both MMSE trajectory plots (left and center) show a generalization of the clusters over time, as the clusters’ order and values are similarly preserved. The time difference with respect to the baseline grows with a consistent steepness (right). Patients belonging to the high-decline cluster (4) present on average a longer time interval between follow-ups.

**Supplemental Table 2.** Clusters summarized by the clustering variables (MMSE score, MMSE decline), and difference of days between the diagnosis and follow-up for the first three follow-ups.

|  | **Cluster (Patients)** | **MMSE score**  **Mean [SD]** | **MMSE decline**  **Mean [SD]** | **Days from diagnosis to follow-up**  **Mean [SD]** |
| --- | --- | --- | --- | --- |
| **Follow-up 1** | Cluster 1 (6031) | 25.03 [2.41] | 1.53 [2.25] | 340.01 [214.72] |
|  | Cluster 2 (4465) | 21.44 [2.01] | -3.18 [1.78] | 392.67 [289.99] |
|  | Cluster 3 (3017) | 16.23 [2.77] | -0.76 [2.10] | 354.50 [217.36] |
|  | Cluster 4 (1915) | 13.09 [3.90] | -8.32 [3.36] | 512.65 [404.30] |
| **Follow-up 2** | Cluster 1 (2108) | 24.62 [2.54] | 0.79 [2.24] | 775.21 [357.04] |
|  | Cluster 2 (1433) | 20.07 [2.35] | -4.31 [1.55] | 799.61 [352.13] |
|  | Cluster 3 (1191) | 16.03 [2.94] | -1.05 [1.83] | 799.27 [335.01] |
|  | Cluster 4 (716) | 11.56 [3.89] | -8.22 [3.09] | 899.69 [411.21] |
| **Follow-up 3** | Cluster 1 (798) | 24.69 [2.50] | 0.43 [1.88] | 1170.73 [381.51] |
|  | Cluster 2 (590) | 19.42 [2.68] | -4.11 [1.49] | 1194.55 [372.62] |
|  | Cluster 3 (481) | 16.16 [2.99] | -0.52 [1.60] | 1218.24 [413.71] |
|  | Cluster 4 (333) | 10.99 [3.75] | -7.41 [2.74] | 1310.30 [405.20] |

**Supplemental Table 3.** Identification of medications statistically associated with the clusters.

| **First follow-up** | | | | | | |
| --- | --- | --- | --- | --- | --- | --- |
| **Drugs** | **p-value; Point estimate, 95% CI (Lower CI – Upper CI)** | | | | | |
|  | **1-2** | **1-3** | **1-4** | **2-3** | **2-4** | **3-4** |
| Atorvastatin |  | 0.026; 1.30 (1.08 – 1.58) | 0.007; 1.44 (1.15 – 1.83) |  |  |  |
| Cyanocobalamin |  | 0.007;0.86 (0.78 – 0.95) |  | 0.001; 0.82 (0.74 - 0.91) |  |  |
| Donepezil | <0.001; 1.21 (1.12-1.31) |  | <0.0001;1.53 (1.37 – 1.71) |  | <0.001; 1.27 (1.13 – 1.42) | <0.0001; 1.33 (1.18 – 1.51) |
| Felodipine |  | 0.002;0.79 (0.66 - 0.95) |  |  |  |  |
| Folic acid |  | <0.001; 0.74 (0.65 – 0.84) |  | <0.001; 0.74 (0.65 – 0.84) |  |  |
| Furosemide |  | <0.001; 0.76 (0.66 - 0.89) |  | <0.001; 0.75 (0.64 – 0.88) |  |  |
| Galantamine |  | <0.001; 1.37 (1.22 – 1.55) | <0.0001; 1.36 (1.18 – 1.56) | <0.001; 1.36 (1.21 – 1.54) | <0.001; 1.34 (1.17 – 1.55) |  |
| Memantine | <0.001;0.78 (0.70-0.86) | <0.001; 0.45 (0.40 – 0.50) | <0.0001;0.44 (0.39 – 0.49) | <0.001; 0.58 (0.52 – 0.64) | <0.001; 0.56 (0.50 – 0.63) |  |
| Oxazepam |  | <0.001; 0.68 (0.57 – 0.81) | <0.0001; 0.60 (0.50 – 0.73) | 0.008; 0.76 (0.64 – 0.91) | <0.001; 0.67 (0.56 – 0.82) |  |
| Paracetamol |  | <0.001; 0.79 (0.70 – 0.88) | 0.022; 0.83 (0.73 – 0.95) | <0.001; 0.79 (0.70 – 0.88) |  |  |
| Rivastigmine |  | 0.003; 1.21 (1.05 – 1.38) |  | <0.001; 1.28 (1.12 – 1.47) |  | 0.012; 0.79 (0.67 – 0.93) |
| Warfarin |  | <0.001; 1.58 (1.28 – 1.97) | <0.001; 1.56 (1.22 – 2.01) | 0.014; 1.37 (1.10 – 1.71) |  |  |
| Zopiclone |  | <0.001; 1.35 (1.15 – 1.58) |  |  |  |  |
| **Second follow-up** | | | | | | |
| **Drugs** | **p-value; Point estimate (%), 95% CI (Lower CI (%) – Upper CI (%))** | | | | | |
|  | **1-2** | **1-3** | **1-4** | **2-3** | **2-4** | **3-4** |
| Atorvastatin |  |  | 0.011; 1.76 (1.20 – 2.64) |  |  |  |
| Galantamine |  | <0.001; 1.44 (1.20 – 1.74) |  | 0.004; 1.46 (1.20 – 1.77) |  |  |
| Memantine | <0.001; 0.72 (0.61 – 0.85) | <0.001; 0.47 (0.40 – 0.55) | <0.001; 0.44 (0.37 – 0.54) | <0.001; 0.65 (0.55 – 0.77) | <0.001; 0.62 (0.51 – 0.75) |  |
| Metformin |  |  | 0.003; 2.08 (1.35 – 3.33) |  |  |  |
| Mirtazapine |  |  | 0.010; 0.66 (0.51 – 0.87) |  |  |  |
| Oxazepam |  | 0.003; 0.63 (0.48 – 0.83) | <0.001; 0.45 (0.33 – 0.61) |  | <0.001; 0.48 (0.35 – 0.67) |  |
| Simvastatin |  |  | 0.015; 1.41 (1.11 – 1.78) |  |  |  |
| **Third follow-up** | | | | | | |
| **Drugs** | **p-value; Point estimate (%), 95% CI (Lower CI (%) – Upper CI (%))** | | | | | |
|  | **1-2** | **1-3** | **1-4** | **2-3** | **2-4** | **3-4** |
| Calcium, vitamin D and others |  |  |  |  | 0.002; 2.56 (1.52 – 4.50) |  |
| Galantamine |  | 0.013; 1.78 (1.32 – 2.41) |  |  |  | 0.019; 0.52 (0.36 – 0.74) |
| Memantine | 0.008; 0.60 (0.47 – 0.76) | <0.001;0.44 (0.34 – 0.57) | <0.001,0.31 (0.23 – 0.41) |  | <0.001;0.51 (0.39 – 0.68) |  |
| Oxazepam |  |  | 0.006; 0.46 (0.28 – 0.74) |  |  |  |

p-value, log odds point estimate, and confidence intervals (CI) in a single simulation for the three follow-ups of significant drugs, same analysis as was shown as a heatmap in Figure 4. Point estimate and CIs are computed by implementing Fisher’s exact test with *scipy* library in Python. The Log odds are computed with respect to the drug consumption proportions in the cluster on the left compared to the cluster on the right. They correspond to the difference of drug usage comparing both proportions. Values greater than one correspond to the cluster on the left presenting a greater proportion. Analogously, a value smaller than one corresponds to the right cluster presenting a greater proportion.

**Supplemental Table 4.** Summary statistics of DDDs taken by medication users, for the selection of medications achieving significant results in clustering analysis

| medication | mean | SD | median | IQR | Dose corresponding to 1 DDD |
| --- | --- | --- | --- | --- | --- |
| Atorvastatin | 1.75 | 1.21 | 1.8 | 1 | 20 mg |
| Cyanocobalamin | 1.16 | 0.43 | 1 | 0 | 1 mg PO |
| Donepezil | 1.15 | 0.59 | 1.3 | 0.68 | 7.5 mg |
| Felodipine | 1.32 | 0.79 | 1 | 0.98 | 5 mg |
| Folic Acid | 7.97 | 7.21 | 3.5 | 10 | 0.4 mg PO |
| Furosemide | 1.19 | 1.01 | 1 | 0.58 | 40 mg |
| Galantamine | 1.07 | 0.61 | 1 | 0.42 | 16 mg |
| Memantine | 0.91 | 0.43 | 1 | 0.48 | 20 mg |
| Metformin | 0.79 | 0.44 | 0.8 | 0.5 | 2 mg |
| Oxazepam | 0.18 | 0.22 | 0.1 | 0.15 | 50 mg |
| Paracetamol | 0.59 | 0.41 | 0.5 | 0.56 | 3 g |
| Rivastigmine | 0.87 | 0.50 | 0.9 | 0.54 | 9 PO / 9.5 TD |
| Simvastatin | 0.96 | 0.51 | 0.7 | 0.67 | 30 mg |
| Warfarin | 0.68 | 0.37 | 0.7 | 0.33 | 7.5 mg |
| Zopiclone | 0.81 | 0.52 | 0.7 | 0.4 | 7.5 mg |
